# Theoretical study of a series of 4,4′-azo-1*H*-1,2,4-triazol-5-one based nitrogen-rich salts as potential energetic compounds†

**DOI:** 10.1039/c7ra13424j

**Published:** 2018-06-29

**Authors:** Wenli Cao, Zimei Ding, Xiaojing Hang, Kangzhen Xu, Jirong Song, Jie Huang, Jiajia Guo

**Affiliations:** School of Chemical Engineering, Shaanxi Key Laboratory of Physico-Inorganic Chemistry, Northwest University Xi'an 710069 China huangjie@nwu.edu.cn; Conservation Technology Department, The Palace Museum Beijing 100009 China

## Abstract

Density function theory has been employed to systemically study 4,4′-azo-1*H*-1,2,4-triazol-5-one (ZTO) and its six nitrogen-rich salts at two different calculated levels (B3LYP/6-31G(d,p) and B3PW91/6-31G(d,p)). Their optimized geometries, electronic structures and molecular electrostatic potentials were further studied. Based on the two computed methods, the results of the optimized geometries show that the calculated structure of each compound adopted at the two different levels are rather similar except salt 7 with some differences. The values of the energy gaps indicate that compound 3 has the highest reactivity among salts 2–7. The crystal densities were corrected using the Politzer approach based on these two optimized levels. The density values with slight deviation indicate that the two calculated levels are applicable and the results are convincible. Based on the isodesmic reactions and Born–Haber energy cycle, the solid-phase heats of formation (HOFs) were predicted. Detonation parameters were evaluated using the Kamlet–Jacobs equations on the foundations of the calculated densities and HOFs. The results manifest that salt 2 exhibits the best detonation performance due to its highest density (1.819 g cm^−3^), followed by salt 6. Moreover, impact sensitivities of compounds 1–7 were assessed using the calculated *Q* values to correlate with *h*_50_. Combining the detonation performance with safety, 1–7 exhibit good comprehensive properties and might be screened as a composition of modern nitrogen-rich energetic compounds.

## Abstract 2

The structural, physicochemical parameters, detonation performances and safeties of 4,4′-azo-1*H*-1,2,4-triazol-5-one and its nitrogen-rich salts were investigated theoretically, and these compounds may serve as modern energetic compounds.

## Graphical abstract

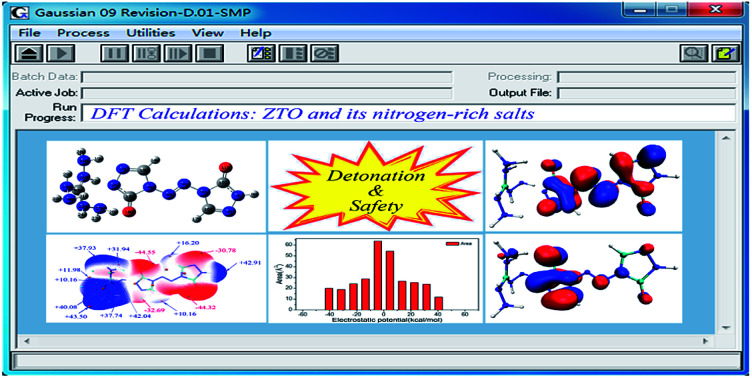

## 1 Introduction

In the development process of modern energetic compounds, higher performance and lower sensitivity continue to be keen concerns.^1–3^ The most desirable characteristics for new energetic compounds include high density, high positive heat of formation, high detonation velocity and pressure, high thermal stability, good oxygen balance and suitable sensitivity. Nitrogen-rich heterocycles, such as, azoles and tetrazines, receive particular interest.^4^ Currently, nitrogen-rich triazolone-based heterocycles are becoming one of the research hot-spots in the field of advanced energetic materials.^5–9^ The introduction of triazolone rings into a molecule can not only generate much environmentally benign molecular nitrogen as the end-product of decomposition, but also change the electronic populations and enhance the aromaticity and stability of the entire molecule.^10–12^ Additionally, these compounds also exhibit many outstanding properties, such as compact structure, high density, high positive formation enthalpy and good kinetic and thermal stability.^1,13–15^ Moreover, the low percentage of carbon and hydrogen in the structure make them easily achieve a good oxygen balance.^16,17^ The above-mentioned features manifest that these heterocycles are expected to be a potential candidate for energetic materials.

In order to explore the potential performances and applications of nitrogen-rich triazolone-based heterocycles, 4,4′-azo-1*H*-1,2,4-triazol-5-one (ZTO) with a huge conjugated system and high symmetrical structure was synthesized by Zhong *et al.*^18^ Later studies found that ZTO was a good energetic building block with excellent thermal stability and a very high nitrogen content (57.15%).^19^ Therefore, ZTO^−^ and ZTO^2−^ are promising to be the candidates of energetic anions. On the other hand, guanidinium, aminoguanidinium, diaminoguanidinium and triaminoguanidinium are important and common nitrogen-rich cations used to construct high-performance energetic compounds. Therefore, the combination of the nitrogen-rich cations and ZTO anions could simultaneously own both high nitrogen concomitant energetic properties and desired remarkable environment compatibility. Moreover, our group has previously reported a series of nitrogen-rich heterocyclic salts as potential energetic materials.^18,20–25^

Given that energetic compounds are to some extent relatively unstable under external stimulus, laboratory studies of these materials may be dangerous. Consequently, theoretically calculating their physicochemical parameters and detonation performances is highly desirable, which makes it possible to screen potential candidates without involving unsafe experimental tests. More importantly, it can also help understand the relationship between molecular structure and property, and which in turn guide the design of energetic materials. In present work, a systematic study on the geometric and electronic structures, molecular electrostatic potentials, densities, heats of formation, detonation properties and impact sensitivities of ZTO and its nitrogen-rich salts were carried out through theoretical methods. Seven compounds including 4,4′-azo-1*H*-1,2,4-triazol-5-one (ZTO, 1),^19,20^ ammonium 4,4′-azo-1*H*-1,2,4-triazol-5-one (A(ZTO), 2),^23^ guanidinium 4,4′-azo-1*H*-1,2,4-triazol-5-one (G(ZTO), 3),^18^ amino-guanidinium 4,4′-azo-1*H*-1,2,4-triazol-5-one (AG(ZTO), 4),^24^ diamino-guanidinium 4,4′-azo-1*H*-1,2,4-triazol-5-one (DAG(ZTO), 5),^24^ triamino-guanidinium 4,4′-azo-1*H*-1,2,4-triazol-5-one (TAG(ZTO), 6),^24^ Bis(guanidinium) 4,4′-azo-1*H*-1,2,4-triazol-5-one (G_2_(ZTO), 7)^25^ were studied.

## 2 Computational methods

Many studies^26–28^ have shown that the density functional theory (DFT) methods, particularly the DFT-B3LYP method with a 6-31G(d,p) basis set, is a credible and widely used approach that not only generates reasonable molecular structures and electron populations, but also gives accurate energies and a series of properties for molecules and ions. However, in most of the equations suggested by Politzer *et al.*^32,33^ in the following sections are parameterized at B3PW91/6-31G(d,p) level. Moreover, these two levels are comparable and generate similar results. Hence, in this article, the geometries of ZTO and all its salts were fully optimized at the B3LYP/6-31G(d,p) and B3PW91/6-31G(d,p) levels with default convergence criteria in Gaussian 09W (Revision D.01) program package respectively.^29^ The harmonic vibrational frequencies were calculated at these two corresponding levels to confirm the structures located at the local minima on the potential energy surfaces without any imaginary frequency. Molecular electrostatic potentials (MEPs) were also obtained using these two respective levels by Multiwfn program,^30^ and the MEP maps were plotted by VMD program.^31^

Crystal density, as the primary and important physical parameter to determine the detonation performance of energetic material, was corrected using a credible method reported by Politzer *et al.* as shown in eqn (1) and (2).^32,33^1
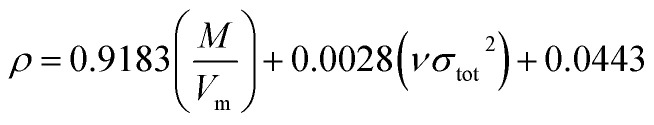
2
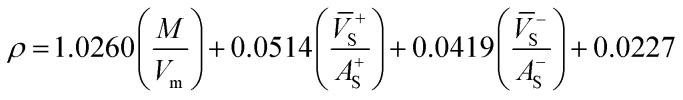
where *M* is the molecular weight in g mol^−1^, *V*_m_ is defined as the volume of the inside of the electron density contour of 0.001 e·bohr^−3^, *ν* indicates the degree of balance between the positive and negative surface potentials, *σ*^2^_tot_ describes the variability of the electrostatic potential on molecule surface, *A*^+^_S_ is the portion of a cation's surface that has a positive electrostatic potential, *V̄*^+^_S_ is the average value of that potential, and *A*^−^_S_ and *V̄*^−^_S_ are the analogous quantities for an anion. The eqn (1) is used to calculate the densities of neutral molecules, while the eqn (2) is an effective option to obtain the densities of energetic ionic compounds.

**Scheme 1:**
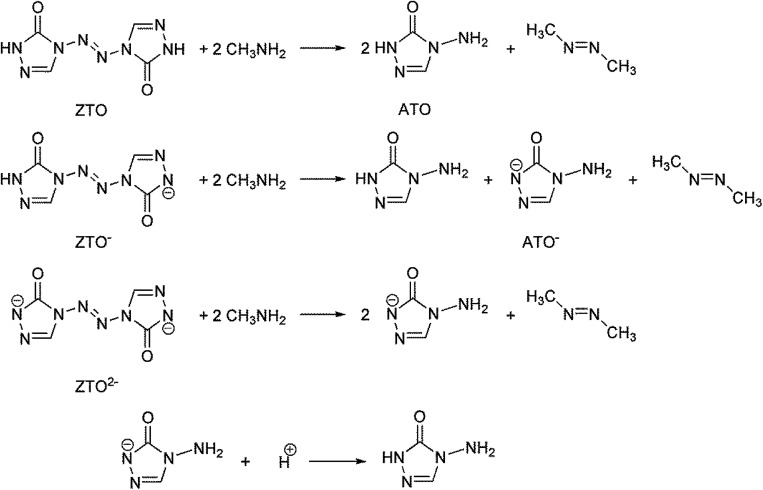
Isodesmic and protonation reactions for calculating the heats of formation.

The heat of formation (HOF) is essential for calculating the detonation performance of the prepared energetic compounds. In order to obtain accurate standard gas-phase HOF 
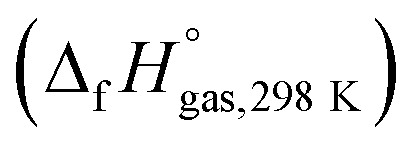
 values, a series of isodesmic reactions were designed to evaluate the HOFs (see Scheme 1). The isodesmic reaction processes, *i.e.*, the number of each kind of formal bond is conserved, are used with application of the bond separation reaction (BSR) rules. The change of enthalpy for an isodesmic reaction at 298 K can be expressed as follows:3
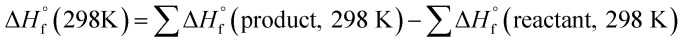
where 
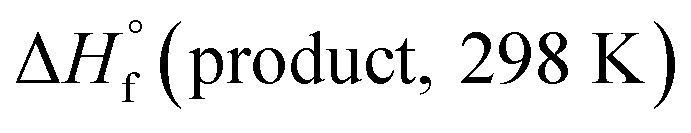
 and 
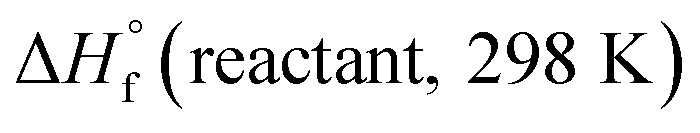
 are the HOF of products and reactants at 298 K, respectively, and 
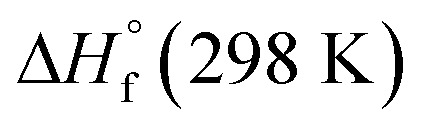
 also can be calculated using the following expression:4
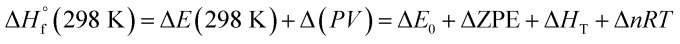
where Δ*E*_0_ is the change in total energy between the products and the reactants at 0 K, ΔZPE is the difference between the zero-point energies (ZPE) of the products and the reactants at 0 K, Δ*H*_T_ is thermal correction from 0 to 298 K, and. the Δ(*PV*) value is the *PV* work term. It equals Δ*nRT* for the reactions of ideal gas. For the isodesmic reactions, Δ*n* = 0, so Δ(*PV*) = 0. On the left side of eqn (3), all the others are called reference compounds except the target compound. The HOFs of reference compounds are available either from the literature 34–36 or from the high level computing like CBS-APNO.^37^

The condensed-phase HOF (Δ*H*_f,s_) of neutral molecule is obtained from the gas-phase HOF (Δ*H*_f,g_) and the heat of sublimation (Δ*H*_sub_) by eqn (5) and (6).5
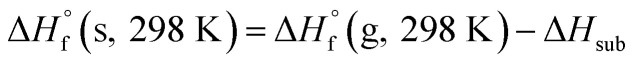
6
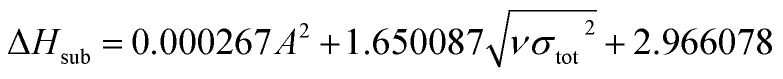
here *A* is the surface area of 0.001 e·bohr^−3^ isosurface of the electronic density, the three coefficients were determined by Rice *et al.*^38^ This method is adopted by many researchers to predict the heat of sublimation of energetic compounds.^39^

**Fig. 1:**
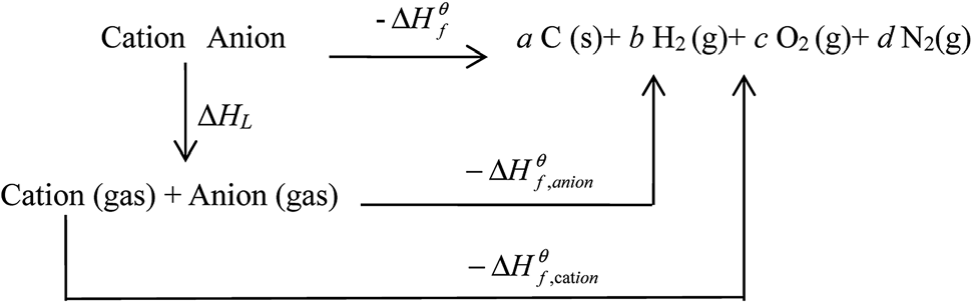
. Born–Haber cycle for the formation for energetic salts.

Based on the Born–Haber energy cycle (see Fig. 1), the HOF of an energetic salt can be expressed as the formula given in eqn (7), in which Δ*H*_L_ is the lattice energy of the salt.7
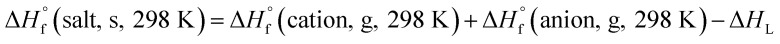


As indicated by the eqn (8), the Δ*H*_L_ could be predicted by the formula proposed by Jenkins *et al.*,^40^ in which *n*_M_ and *n*_X_ depend on the nature of the ions M^p+^ and X^q−^, respectively, and are equal to 3 for monatomic ions, 5 for linear polyatomic ions and 6 for nonlinear polyatomic ions.8
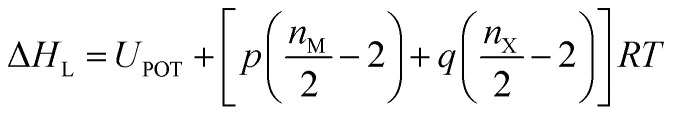


The lattice potential energy (*U*_POT_), is expressed as follows (eqn (9)):

9 *U*_POT_ = *α*(*ρ*/*M*)^1/3^ + *β*

there *ρ* is the density in g cm^−3^, *M* is the chemical formula mass of the ionic material in g. *α* and *β* are coefficients, whose values are 1981.2 kJ mol^−1^ cm and 103.8 kJ mol^−1^ for compound like MX (1 : 1), and 8375.6 kJ mol^−1^ cm and −178.8 kJ mol^−1^ for compound like M_2_X (1 : 2), respectively.^40^

The detonation parameters such as detonation pressure (*P*), detonation velocity (*D*) and heat of detonation (*Q*) can evaluate the energy level of an energetic compound. Based on the densities and calculated HOFs for the title compounds, the *P* and *D* were calculated according to the Kamlet–Jacobs equations for a molecular formula form like C_*a*_H_*b*_O_*c*_N_*d*_.^41^

10 *D* = 1.01(*NM̄*^0.5^Q^0.5^)^0.5^(1 + 1.30*ρ*)

11 *P* = 1.558*ρ*^2^*NM̄*^0.5^Q^0.5^

where *N* is the moles of detonation gases per gram explosive (mol g^−1^); *M̄* is the average molecular weight of these gases (g mol^−1^); *Q* is the heat of detonation (cal g^−1^) and denotes the total heat release in a detonation reaction per gram of an energetic compound, which should be calculated before *D* and *P* are determined; and *ρ* is the loaded density of explosives (g cm^−3^). The measured densities were used for the calculation here. Table 1 presents the methods for calculating the *N*, *M̄*and *Q* parameters of the C_*a*_H_*b*_O_*c*_N_*d*_ compounds.

**Table 1:** Calculated methods for the values of *N*, *M̄*and *Q* of C_*a*_H_*b*_O_*c*_N_*d*_ compounds^a^

	*N*	*M̄*	*Q* × 10^−3^
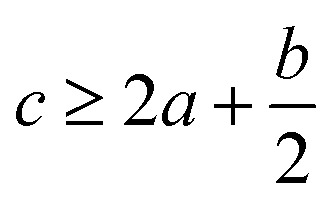	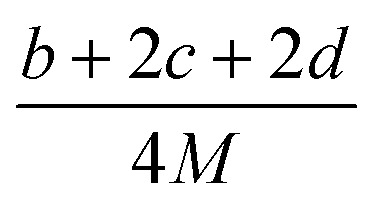	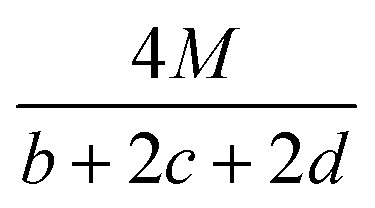	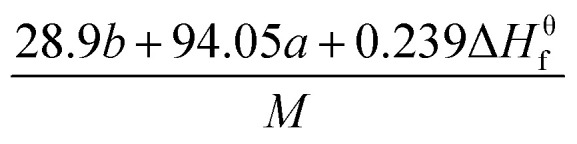
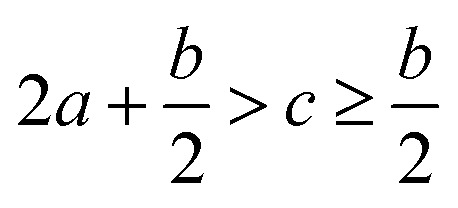	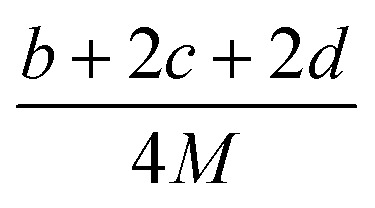	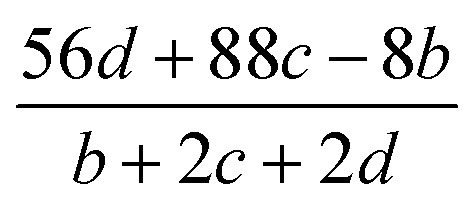	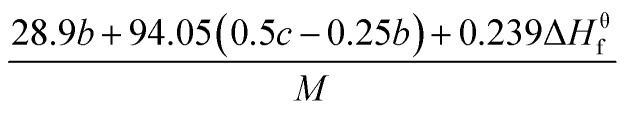
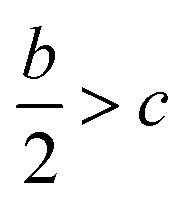	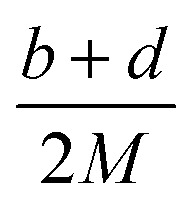	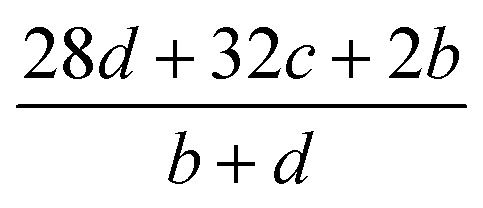	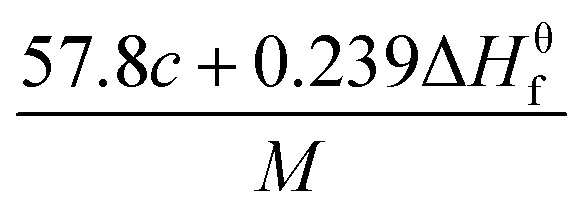

a *M* is the molecular weight in g mol^−1^; Δ*H*^θ^_f_ is the solid phase HOF in kJ mol^−1^.

## 3 Results and discussion

### 3.1 Optimized geometries

**Fig. 2:**
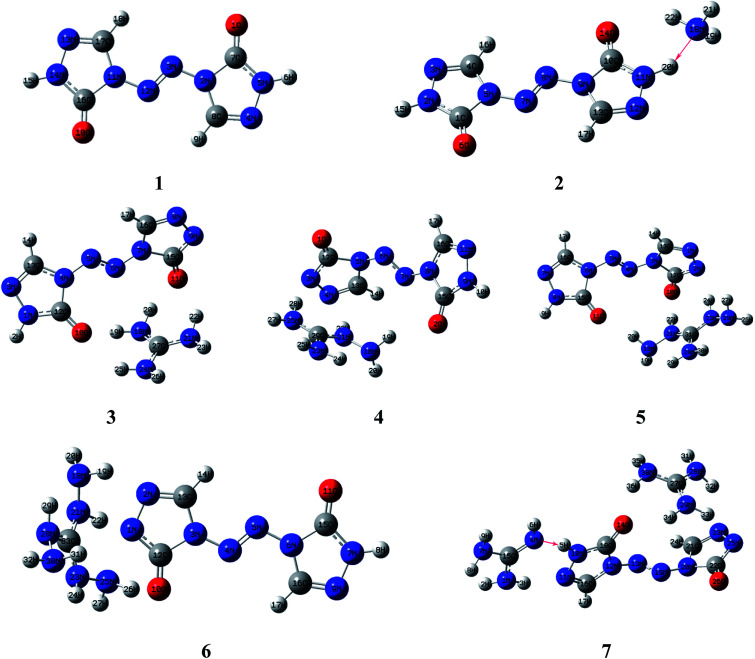
. Optimized geometries of 1–7 by DFT-B3LYP/6-31G(d,p) level.

Molecular structures of the seven compounds were extracted from the single crystal X-ray structures to be used as their starting geometries for geometry optimization. The starting geometries of compounds 1–7 were shown in Fig. S1 (see ESI†). The optimized geometries of them by DFT-B3LYP/6-31G(d,p) level of theory were demonstrated in Fig. 2. And their optimized geometries at DFT-B3PW91/6-31G(d,p) level were also plotted as shown in Fig. S2 (see ESI†). The results show that the optimized geometries of 1–6 at B3LYP/6-31G(d,p) level are rather similar to that of the B3PW91/6-31G(d,p) level. Comparing optimized geometries with experimental molecular structures, the planar structures of ZTO ions are destroyed in salts 3–5 at these two levels. These mentioned deviations could be attributed to the different physical states between the measured (solid) and the calculated (gas). It is also worth mentioning that the proton transfer processes occurred between anions and the corresponding cations in salts 2 and 7 after optimization (see Fig. 2 and S2†), which is also mentioned in the literature 42. There are still some differences between the two computed methods. Like compound 7, the optimized geometry changes from symmetrical to asymmetrical form and the coplanar structure of the anion ZTO^2−^ is destroyed at B3LYP/6-31G(d,p) level. However, the optimized conformation with the perfectly symmetrical structure is almost consistent with the starting geometry at B3PW91/6-31G(d,p) level. So, in this study, we suspect that the optimized geometries computed by B3PW91/6-31G(d,p) level are more reliable than that of the B3LYP/6-31G(d,p) level.

### 3.2 Electronic structures

Molecular orbital analysis could provide valuable information on its electronic structure, therefore the frontier molecular orbital (FMO) theory has been broadly used by chemists. The HOMO–LUMO gap between the highest occupied molecular orbital (HOMO) and the lowest unoccupied molecular orbital (LUMO) can be correlated with the sensitivity of material within the limitations of DFT.^43^ In general, the smaller the HOMO–LUMO gap is, the easier the electron transition is, the lower the kinetic stability is.^44–46^ Besides, the FMO theory plays a significant role in electronic, electric and optical properties as well as in the quantum chemistry. The LUMO as an electron acceptor denotes the capability to obtain an electron, while HOMO as an electron donor gives away an electron.^47^

**Fig. 3:**
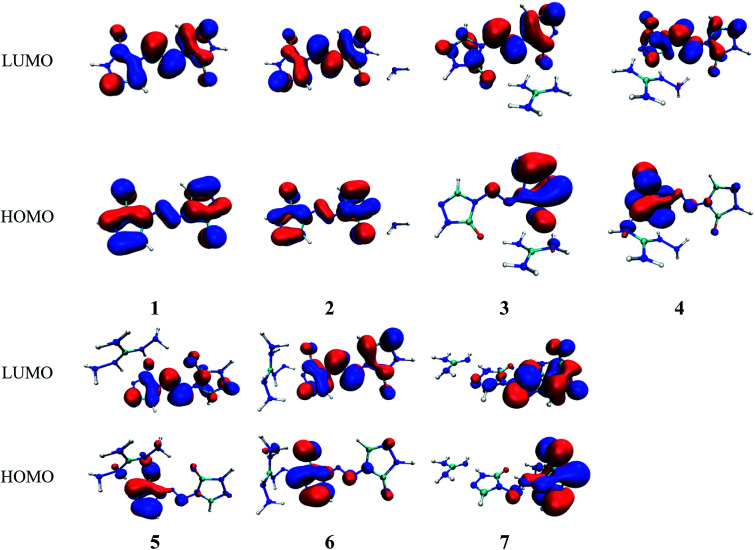
The pictorial illustration of HOMO and LUMO at B3LYP/6-31G(d,p) level for 1–7.

**Fig. 4:**
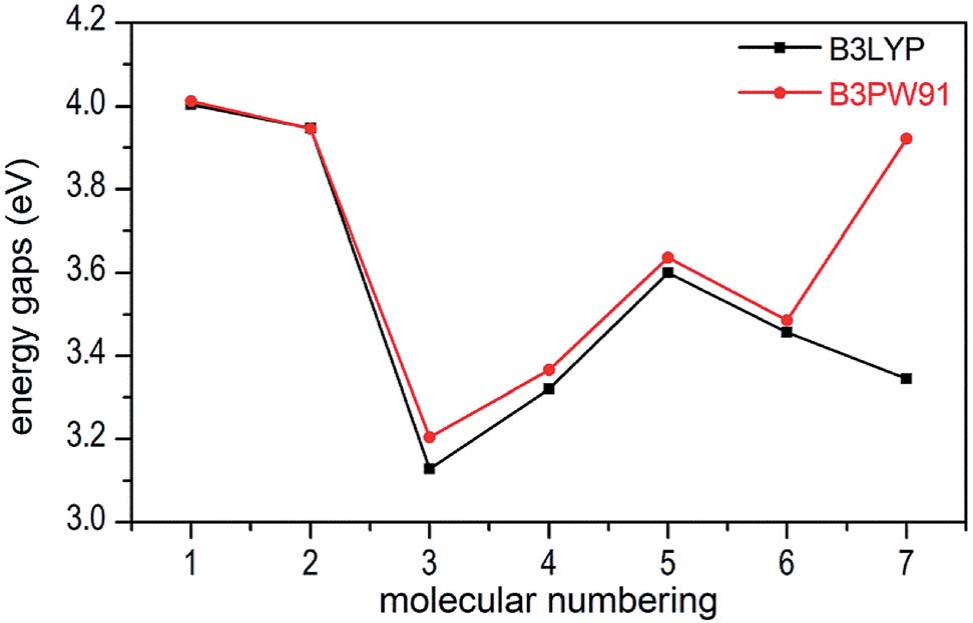
Comparison of the energy gaps (Δ*E*_LUMO–HOMO_) for 1–7 with different levels.

In present work, the HOMO–LUMO gaps of 1–7 were predicted using DFT at B3LYP/6-31G(d,p) and B3PW91/6-31G(d,p) levels. The HOMO and LUMO energies and their energy gaps (Δ*E*_LUMO–HOMO_) were listed in Table 2. The diagrams of HOMO and LUMO at B3LYP/6-31G(d,p) level for 1–7 were plotted by VMD and shown in Fig. 3. The diagrams of HOMO and LUMO at B3PW91/6-31G(d,p) level for compound 7 was shown in Fig. S3.† All the HOMO and LUMO isosurfaces were mapped for an isovalue 0.03. The red and blue colors of the isosurfaces represent lobes of positive and negative phase wave function, respectively. Fig. 4 presents a comparison of the energy gaps (Δ*E*_LUMO–HOMO_) for 1–7 with the two different levels.

**Table 2:** Calculated HOMO and LUMO energies (eV) and energy gaps (Δ*E*_LUMO-HOMO_) of 1–7

Comp	*E*_HOMO_		*E*_LUMO_		Δ*E*_LUMO-HOMO_	
	B3LYP	B3PW91	B3LYP	B3PW91	B3LYP	B3PW91
1	−6.598	−6.675	−2.594	−2.663	4.004	4.012
2	−6.323	−6.358	−2.376	−2.412	3.947	3.946
3	−5.175	−5.205	−2.046	−2.001	3.129	3.204
4	−5.285	−5.327	−1.964	−1.962	3.321	3.365
5	−5.432	−5.536	−1.832	−1.899	3.600	3.637
6	−5.161	−5.248	−1.705	−1.763	3.456	3.485
7	−4.985	−5.995	−1.640	−2.073	3.345	3.922

As shown in Fig. 3, the electronic clouds mainly focus on the ZTO anions but almost none on cations on both HOMO and LUMO for compounds 1–7. The distribution of electronic clouds in compound 1 is exactly similar to that in compound 2. Moreover, the LUMOs are largely localized almost over the whole ZTO anions whereas the region of HOMOs spread approximately half of the entire ZTO anions for compounds 3–6 at B3LYP/6-31G(d,p) level. Similarly, compound 7 presents different distribution of HOMO and LUMO due to the different optimized geometries under the two methods (see Fig. 3 and S3†).

From Table 2, we found that the energy gaps of 1–7 at B3PW91/6-31G(d,p) level are higher than that of the B3LYP/6-31G(d,p) level except compound 2. Moreover, the energy gaps of all salts are smaller than that of their precursor 1, which indicates that the kinetic stability is reduced by salt-forming reaction. Among these salts, the Δ*E*_LUMO–HOMO_ value of 2 was the highest, whereas the one for 3 was the lowest. Furthermore, since the molecule with smaller HOMO–LUMO gap was expected to have higher reactivity and lower kinetic stability in the chemical reactions with electron transfer,^45,46^ it might be inferred that compound 3 had the highest reactivity among these salts and it can react with nitrogen-rich salts by metathesis reaction to obtain other promising salts of ZTO with higher nitrogen content. It is remarkable that the Δ*E*_LUMO–HOMO_ values of compound 7 show a great variation at the two levels, as shown in Table 2 and Fig. 4.

### 3.3 Molecular electrostatic potentials

**Fig. 5:**
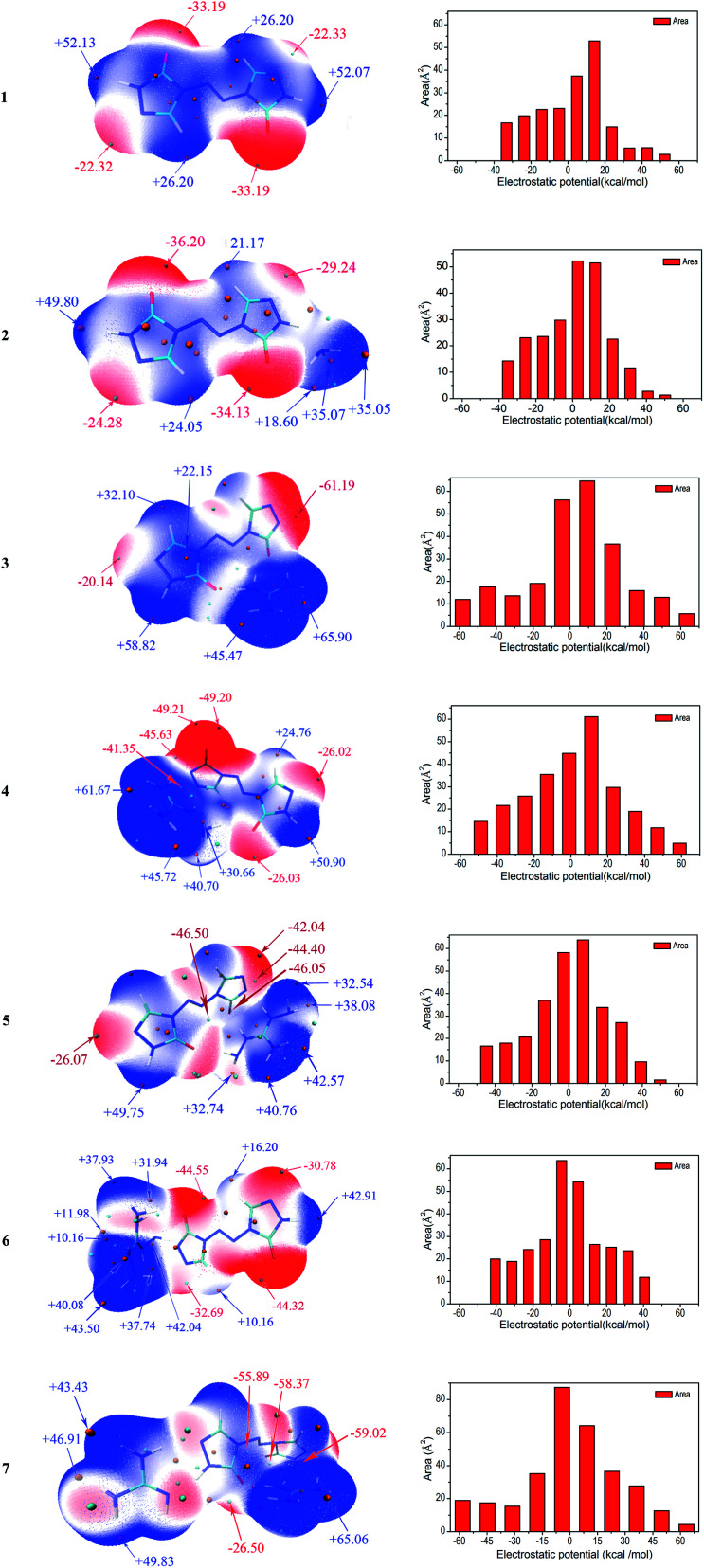
(contd.)

Molecular electrostatic potentials (MEPs, *V*(*r*)) have been widely used for prediction of nucleophilic and electrophilic sites for a long time. The theoretical basis is that molecules always tend to approach each other in a complementary manner of MEP.^30^ On the other hand, the molecular electrostatic potential parameters are always served to predict the impact sensitivity of CHNO energetic compounds.^48,49^ These analyses of MEPs are commonly performed on molecular van der Waals (vdW) surface. MEPs for the optimized geometries were calculated using Multiwfn program for 1–7. The selected electrostatic potential parameters of compounds 1–7 were shown in Table S1 (see ESI†). Their MEP maps at B3LYP/6-31G(d,p) level were plotted by VMD shown in Fig. 5. The color scale is in the range from −25 to 25 kcal mol^−1^. The red color represents negative electrostatic potential while the blue one indicates positive electrostatic potential, and these white ones represent zero potential. Meanwhile, the surface extrema were labeled, and the orange and cyan spheres stand for maxima and minima, respectively.

Through comparing the surface area of two methods, as shown in Table S1,† the positive regions (*A*^+^_S_) cover a larger portion of the total surface area than the negative ones (*A*^−^_S_) for 1–5, which is exactly consistent with the electrostatic potential distribution of energetic systems proposed by Hammerl *et al.*^50^ In this section, we take the results at B3LYP/6-31G(d,p) level as an example to understand the molecular electrostatic potentials for 1–7. For the seven compounds, the minima of the MEPs appear near the oxygen atoms in the carbonyl groups, as the electron-withdrawing groups, while the maxima tend to occur at hydrogen atoms in the –NH_2_ groups in cations and –NH– groups of ZTO anions. The global maximum of the MEPs corresponding to the hydrogens for compounds 3–6 are +65.90, +61.67, +49.75 and +43.50 kcal mol^−1^, while the minimum corresponding to the oxygens are −61.19, −49.21, −46.50 and −44.55 kcal mol^−1^, respectively. It is well-known that the minima and maxima are relatively reactive sites for nucleophilic and electrophilic attack, respectively. As a consequence, salts 3 and 7 can react with nitrogen-rich salts to obtain other valuable salts of ZTO with better performances, which is well consistent with our previous analysis in Section 3.2. The area for each MEP range in Fig. 5 can provide more information. From the prediction, the values of the MEPs are mainly distributed in the range from −50 to +50 kcal mol^−1^ except 3 and 7 ranging from −60 to 70 kcal mol^−1^. The largest areas have values located near to +10 kcal mol^−1^ except 6 and 7 of about −5 kcal mol^−1^. The areas with values larger than +30 kcal mol^−1^ should consist of the hydrogen atoms in amino groups and –NH– groups of triazolone rings.

### 3.4 Densities

A good density is one of the most attractive properties for energetic materials, which is directly influence on the detonation performance. The densities of the five compounds 2–6 were estimated by eqn (2) while the density of 1 was calculated using eqn (1). According to the requirements of the eqn (2), the *V*_m_ was taken to be the sums of the volumes of the ions comprising a formula unit of the compound, and the volumes were defined by the 0.001 e·bohr^−3^ contours of the ions' electronic densities. The data of electrostatic potential parameters of ions used by density calculation were summarized in Table S2.† The values of molecular weight (*M*), total volume (*V*_m_) and density (*ρ*) for compounds 1–7 are listed in Table 3.

**Table 3:** The values of *M*, *V*_m_ and *ρ* for compounds 1–7

	*M*/g mol^−1^	*V*_m_/Å^3^		*ρ*_cal_/g cm^−3^	
		B3LYP	B3PW91	B3LYP	B3PW91
1	196.13	191.17	190.93	1.766	1.775
2	213.16	222.94	223.67	1.819	1.816
3	255.20	269.28	270.48	1.687	1.680
4	270.21	284.85	286.05	1.675	1.668
5	285.23	301.49	302.11	1.662	1.659
6	300.24	316.25	317.26	1.661	1.653
7	314.31	349.24	349.58	1.640^a^	

a The value was obtained from X-ray data.

**Fig. 6:**
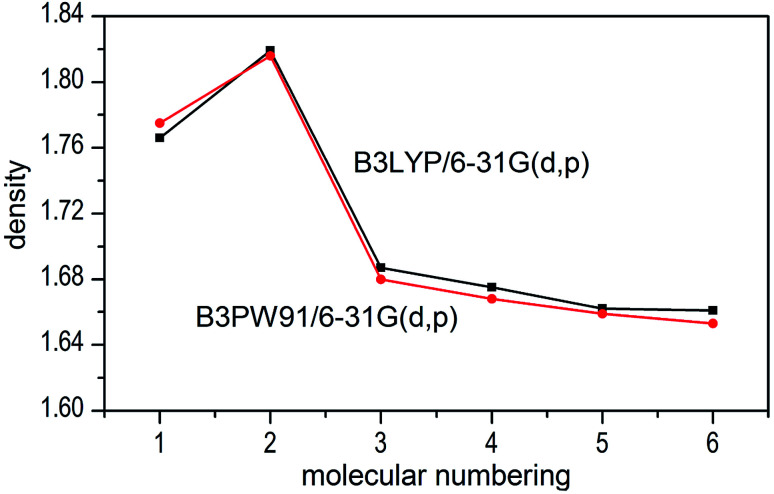
Comparison of the density for 1–7 at the two different levels.

The densities of 1–6 show that the values obtained by B3LYP/6-31G(d,p) level are very close to that computed by B3PW91/6-31G(d,p) level with the maximum deviation of only 0.009 g cm^−3^ (see Fig. 6), which manifest that the two calculated levels are applicable and the results are convincible. It can be inferred that the results of detonation parameters may be basically consistent because of the slight deviation (0.009 g cm^−3^ < 0.03 g cm^−3^)^51^ at the two levels. Take the first method as an example, compounds 1–7 exhibit good densities ranging from 1.640 g cm^−3^ to 1.819 g cm^−3^, which are comparable to the currently used energetic materials (EMs, 1.60–1.80 g cm^−3^).^52^ It is noteworthy that compound 2 possesses a relatively high density (1.819 g cm^−3^) to the level of new high-energy-density materials (HEDMs, 1.80–2.0 g cm^−3^).^52^ Hence, we believe that compound 2 may show the best detonation properties among these compounds. It is generally true that salt formation results in a lower density, so in this paper all the salts 3–7 have lower densities than their precursor of ZTO (1.766 g cm^−3^) except salt 2.

### 3.5 Heats of formation

Heats of formation (HOFs) are another important property to evaluate the performances of the prepared energetic salts. The gas-phase HOF at 298 K of ZTO was calculated according to the isodesmic reaction illustrated in Scheme 1. The enthalpy of an isodesmic reaction is obtained by combining the total energy difference for the reaction, the scaled zero-point energies and other thermal factors.

The experimental HOFs of reference compounds CH_3_NH_2_ and CH_3_N=NCH_3_ were taken from the literatures 34–36. Since the experimental HOF of the reference compound 4-amino-1,2,4-triazol-5-one (ATO) is unavailable, additional calculation was carried out for the atomization reaction: C_2_H_4_N_4_O → 2C (g) + 4H (g) + 4N (g) + O (g) at the CBS-APNO level to obtain its HOF which is 103.60 kJ mol^−1^. To validate the reliability of our calculation results, the HOFs of molecules CH_3_NH_2_ and CH_3_N=NCH_3_ were also calculated from the atomization reaction at the CBS-APNO theory level. The results show that their HOF values are very close to the corresponding experimental values with the relative errors of only 3.92% and 1.86%, respectively. Therefore, the HOF values from the CBS-APNO calculations are expected to be reliable in the present study. Similarly, the HOFs of the anions of ZTO^−^ and ZTO^2−^ were calculated by the same procedure using isodesmic reactions given in Scheme 1. The HOF of the ATO^−^ anion was calculated according to the protonation reaction shown in Scheme 1 to be 45.22 kJ mol^−1^. Table 4 lists the total energies, ZPEs, thermal corrections and HOFs for reference compounds and target compounds in the isodesmic reactions.

**Table 4:** Calculated total energies (*E*_0_), zero-point energies (ZPEs), thermal corrections (*H*_T_) and heats of formation (HOFs) for the reference compounds and target compounds in the isodesmic and protonation reactions^a^

Compound	*E*_0_/a.u.	ZPE/a.u.	*H*_T_/kJ mol^−1^	HOF/kJ mol^−1^	HOF^c^/kJ mol^−1^
CH_3_NH_2_	−95.842437	0.0642	11.45	−23.38^b^	−22.50
	−95.842478	0.0644	11.45	−23.38^b^	
CH_3_N=NCH_3_	−189.254192	0.0845	16.10	154.63^b^	151.80
	−189.254392	0.0847	16.08	154.60^b^	
ATO	−372.811031	0.0809	18.73	103.60^b^	
	−372.811523	0.0816	18.53	103.40^b^	
ATO^−^	−372.236582	0.0661	18.52	45.22^d^	
	−372.237215	0.0668	18.33	44.86^d^	
ZTO	−743.205429	0.1175	32.34	367.74^e^	
	−743.206434	0.1186	31.97	367.39^e^	
ZTO^−^	−742.661757	0.1030	31.97	229.19^e^	
ZTO^2−^	−742.015657	0.0897	30.72	355.63^e^	
H^+^	0	0	6.196	—	1536.20

a The values of the previous line were obtained based on the optimized geometries at B3LYP/6-31G(d,p) level while the second lines corresponding to the B3PW91/6-31G(d,p) level. *E*_0_ and ZPE are in a.u., and the *E*_0_ were calculated at M062X/def2tzvp level; *H*_T_ and HOF are in kJ mol^−1^. The scaling factor is 0.9806 for ZPE.^53^

b The values were calculated at the CBS-APNO level.

c The experimental HOFs were taken from literatures 34–36, respectively.

d The HOF of ATO^−^ was calculated from the protonation reaction.

e The values were obtained from the isodesmic reactions.

In order to explore the differences of the HOFs based on the two different optimization methods (B3LYP/6-31G(d,p) and B3PW91/6-31G(d,p) level), the HOFs of ZTO and selected reference compounds were also estimated based on B3PW91/6-31G(d,p) level. These results are shown in Table 4. The results of HOF at the two optimization methods further indicate that these tiny differences are not so important for optimized levels in the present work. In addition, a density change of 0.1 g cm^−3^ significantly impacted on the explosive performance, while a difference of 10 kcal mol^−1^ in HOF had little influence.^51^ Therefore, it is reasonable to believe that the calculated methods are applicable and the results are reliable. And all the subsequent computations will be done using the values on the basis of B3LYP/6-31G(d,p) level. Namely, the scaled zero-point energies and other thermal factors were obtained from the optimized geometries using B3LYP/6-31G(d,p) level, and the total energies (single-point energies) were calculated by M062X/def2tzvp method for the isodesmic reaction.

From the Table 4, the gas-phase HOF of ZTO (367.74 kJ mol^−1^) is much larger than that of ATO (103.60 kJ mol^−1^), which indicates that the –N=N– bridge group is a very excellent linkage for increasing HOFs for these ZTO-based derivatives. And this change was also observed in other research papers.^54^ Together with the heat of sublimation (Δ*H*_sub_) given by eqn (6) using molecular electrostatic potential parameters, the condensed-phase HOF (Δ*H*_f,s_) of ZTO is estimated according to eqn (5).

The gas-phase HOFs of the cations were obtained from the ref. 13, 59 and 60, respectively. And then, the results which the solid phase HOFs of the ZTO-based salts 2–7 were calculated based on the Born–Haber energy cycle are shown in Table 5. As currently used classical explosives, 2,4,6-trinitrotoluene (TNT), hexahydro-1,3,5-trinitro-1,3,5-triazine (RDX) and octahydro-1,3,5,7-tetranitro-1,3,5,7-tetrazocine (HMX) are extensively used as reference compounds to evaluate the performance of the new designed compounds. Their properties are also shown in Table 5. From the table, we can see that all the six salts exhibit high positive heats of formation for solid phase ranging from 228.57 kJ mol^−1^ (7) to 641.53 kJ mol^−1^ (6), which are higher than that of TNT (−67.7 kJ mol^−1^),^55^ RDX (92.6 kJ mol^−1^)^56^ and HMX (104.8 kJ mol^−1^).^56^ Because of the high lattice energy of the salts (Δ*H*_L_, see Table 5), the values of Δ_f_*H*_salt_ decreased evidently compared to the sums of Δ_f_*H*_cation_ and Δ_f_*H*_anion_. It is worth noting that the heat of formation of G_2_(ZTO) (7) is relatively lower than that of ZTO (1) and other salts, which may be explained by the fact that G_2_(ZTO) possesses the highest the lattice energy (Δ*H*_L_) among all guanidine salts. The HOF of guanidinium cations gradually increased with the increasing of amino group in compounds 3–6, from which we can infer that amino group may be an excellent group for markedly improving the HOF of energetic compound. Consequently, the HOFs of compounds 3–6 are sequenced as 3 < 4 < 5 < 6, which also indicate that the family of guanidinium ions are outstanding nitrogen-rich cations used to construct high-performance energetic compounds. Especially, compound 6 possesses the highest heat of formation for solid phase among these compounds.

Energetic properties of compounds 1–7

**Table:** 

Comp	N^a^%	OB^a^	*ρ*_cal_ b/g cm^−3^	Δ*H*_f,g_/kJ mol^−1^	A/Å^2^	ν*σ*_tot_^2^/(kcal mol^−1^)^2^	Δ*H*_sub_^c^/kJ mol^−1^	Δ*H*_f,s_/kJ mol^−1^	Q^d^/cal g^−1^	P^j^/GPa	D^k^/ms^−1^
1	57.15	−32.6	1.766	367.74	210.295	56.113	113.53	254.21	899.53	22.14	7106

a Nitrogen content and oxygen balance, for the compound with the molecular formula of C_*a*_H_*b*_N_*c*_O_*d*_, OB = 1600[(*d* − *a* − *b*/2)/*M*].

b Calculated density except 7 obtained from X-ray data.

c Heat of sublimation.

d Heat of detonation.

e Calculated enthalpy of formation of cations, ref. 13, 59 and 60.

f Calculated molar enthalpy for the formation of the anion.

g Lattice potential energy.

h The lattice energy of the salts.

i Calculated molar enthalpy for the formation of the salts.

j Detonation pressure.

k Detonation velocity.

l From ref. 58.

m From ref. 55.

n From ref. 56.

**Table:** 

Comp	N^a^%	OB^a^	*ρ*_cal_ b/g cm^−3^	Δ_f_*H*_cation_^e^/kJ mol^−1^	Δ_f_*H*_anion_^f^/kJ mol^−1^	*U*_POT_ g/kJ mol^−1^	Δ*H*_L_^h^/kJ mol^−1^	Δ_f_*H*_salt_^i^/kJ mol^−1^	Q^d^/cal g^−1^	P^j^/GPa	D^k^/ms^−1^
2	59.14	−41.3	1.819	626.40	229.19	482.19	487.14	368.45	955.82	27.16	7801
3	60.37	−47.0	1.687	575.90	229.19	475.63	480.58	324.51	757.19	21.11	7037
4	62.20	−47.4	1.675	667.40	229.19	467.74	472.70	423.89	803.08	22.03	7205
5	63.84	−47.7	1.662	769.00	229.19	460.31	465.27	532.92	852.19	22.89	7362
6	65.31	−47.9	1.661	871.50	229.19	454.20	459.16	641.53	896.09	23.94	7530
7	62.40	−56.0	1.640	1151.80	355.63	1273.91	1278.86	228.57	541.80	18.06	6568
TNT	18.5	−74	1.65^l^					−67.0^m^	1290^l^	19.53^m^	6881^m^
RDX	37.8	−21.6	1.81^l^					92.6^n^	1500^l^	34.9^n^	8748^n^
HMX	37.8	−21.6	1.89^l^					104.8^n^	1500^l^	39.2^n^	9059^n^

### 3.6 Detonation properties and impact sensitivities

**Fig. 7:**
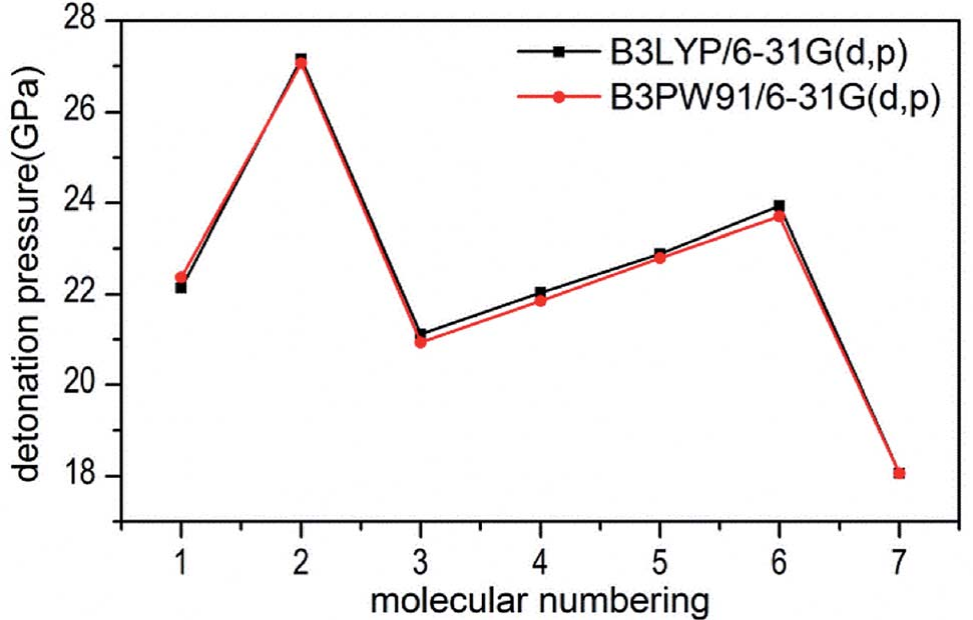
Comparison of P for 1–7 based on different density values obtained by the two different levels.

**Fig. 8:**
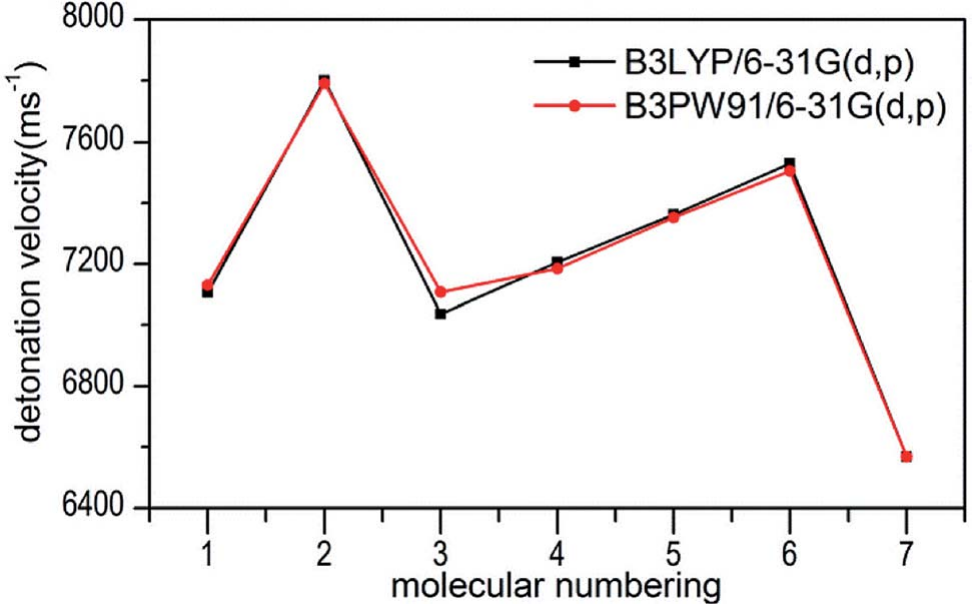
Comparison of *D* for 1–7 based on different density values obtained by the two different levels.

Several key parameters of explosive performance are the heat of detonation (*Q*), detonation velocity (*D*) and detonation pressure (*P*). *Q*, *D* and *P* can be evaluated by Kamlet–Jacobs empirical equations (see Table 1 and eqn (10) and (11)) based on their density and calculated solid-phase heats of formation. *D* is proportional to the molecular density, while P is proportional to the square of the molecular density. The explosive reaction was identified by applying the “most exothermic” principle. Namely, all of the N atoms turn into N_2_, while the O atoms initially react with H atoms to give H_2_O before forming CO_2_ with the C atom. So the heat of detonation refers to the maximum heat of detonation in present work. The calculated results of *Q*, *D* and *P* based on optimized geometries at B3PW91/6-31G(d,p) level are summarized in Table 5. The results of *P* and *D* based on different density values corresponding to B3PW91/6-31G(d,p) level are listed in Table S3.† The results show that the values of *P* and *D* based on different density values are very close when the values of HOF remain unchanged (Fig. 7 and 8), which is exactly agreement with our previous prediction in Section 3.4. And all the subsequent discussion will be performed using the computed values based on B3LYP/6-31G(d,p) level.

The calculated values of *Q* for 1–7 lie between 541.80 cal g^−1^ (7) and 955.82 cal g^−1^ (2). The detonation pressures of the synthesized energetic salts were found to be in the range of 18.06 GPa (7) to 27.16 GPa (2) and their detonation velocities are between 6568 m s^−1^ (7) and 7801 m s^−1^ (2), all of which outperform the classical explosive TNT (except 7) but still lower than that of RDX and HMX. Among these compounds, the ammonium 4,4′-azo-1*H*-1,2,4-triazol-5-one (2) exhibit the highest detonation performances apparently because of its highest density, which is perfectly consistent with our previous prediction in Section 3.4. The relatively high detonation performances of the triamino-guanidinium 4,4′-azo-1*H*-1,2,4-triazol-5-one (6) should be attribute to its highest positive heat of formation and good density. The third high detonation performances of the diamino-guanidinium salt (5) may be the result of the same factors with compound 6, while the 4,4′-azo-1*H*-1,2,4-triazol-5-one (1) exhibit good detonation performances because of its higher density than others. Oxygen balance (OB) is an expression that indicates the degree to which an explosive can be oxidized.^57^ All the compounds in this study have negative OBs ranging from −56.0% (7) to −32.6% (1). Besides, all of them show high nitrogen content between within 57.17% (1) to 65.31% (6), which are significantly greater than that of TNT (18.5%) and RDX (37.8%).

Impact sensitivity (*h*_50_) is one of key properties to judge the operational safety of an energetic compound, and it also reflects the ease of initiating detonation of energetic materials. In recent reports, Politzer and Murray correlated the maximum heat of detonation (*Q*) with the impact sensitivity (*h*_50_), which reveals that higher values of *Q* refer to greater impact sensitivity (*h*_50_) and this conclusion can be applicable to all types of explosives.^58^ Combined with the calculated *Q* values of 1–7, the salt 2 will presents the highest impact sensitivity owing to its highest *Q* value while salt 7 should be possesses the lowest one. In addition, salts 3–7 are more insensitive than their precursor 1. Remarkably, all of the compounds 1–7 are expected to be insensitive compared with TNT, RDX and HMX.

According to our previous studies, the decomposition temperatures of these compounds are above 200 °C, which perfectly supports their thermal stability.^18,24^ And considering its good detonation properties, these compounds could be considered as the potential candidates of energetic materials.

## 4 Conclusion

In this work, the properties of 4,4′-azo-1*H*-1,2,4-triazol-5-one (1) and its nitrogen-rich salts 2–7 previously reported have been studied theoretically. Their starting geometries were extracted from the single crystal X-ray data and fully optimized at two different calculated levels (B3LYP/6-31G(d,p) and B3PW91/6-31G(d,p)) respectively. Through DFT calculation, their optimized geometries, electronic structures, molecular electrostatic potentials and many properties such as densities, heats of formation, detonation properties and impact sensitivities were further investigated. The results show that the optimized geometries at B3LYP/6-31G(d,p) level are very similar to that of the B3PW91/6-31G(d,p) level except salt 7. The energy gaps manifest that salt 3 possesses the highest reactivity among these salts and can react with nitrogen-rich salts to obtain other potential ZTO-based salts with better properties. All compounds exhibited good densities of 1.640 g cm^−3^ (7) to 1.819 g cm^−3^ (2) on the basis of B3LYP/6-31G(d,p) level. The HOF of ZTO was predicted to be 254.21 kJ mol^−1^ based on the designed isodesmic reactions. The HOFs of salts 1–7 were calculated based on the Born–Haber cycle ranging from 228.57 kJ mol^−1^ (7) to 641.53 kJ mol^−1^ (6), which are higher than that of TNT, RDX and HMX. Their detonation velocities and detonation pressures were evaluated to be in the range of 6568 m s^−1^ (7) to 7801 m s^−1^ (2) and 18.06 GPa (7) to 27.16 GPa (2), respectively. The calculated values of *Q* for 1–7 lie between 541.80 cal g^−1^ (7) and 955.82 cal g^−1^ (2). Therefore salt 2 exhibits the highest impact sensitivity while salt 7 should be the lowest one. In addition, salts 3–7 are more insensitive than their precursor 1. Remarkably, all of the compounds 1–7 are expected to be insensitive compared with TNT, RDX and HMX. Considering the thermal stability, detonation performance and safety, these compounds could be screened as a composition of modern nitrogen-rich energetic materials. This work provides valuable information for the preparation of novel ZTO-based high performance energetic compounds.

## Conflicts of interest

There are no conflicts to declare.

## Supplementary Material

RA-008-C7RA13424J-s001
