# Supplementary material for: Theoretical study of a series of 4,4′-azo-1H-1,2,4-triazol-5-one based nitrogen-rich salts as potential energetic compounds
Source: RSC Adv. 2018 Jun 29;8(42):23805–16. doi: 10.1039/c7ra13424j (PMC9081749; doi:10.1039/c7ra13424j)
Supplement: RA-008-C7RA13424J-s001 [file RA-008-C7RA13424J-s001.pdf]

Electronic Supplementary Material (ESI) for RSC Advances.
This journal is © The Royal Society of Chemistry 2018

Electronic Supporting Information (ESI)

Theoretical study of a series of 4,4′-Azo-1*H*-1,2,4-triazol-5-one based nitrogen-rich salts as potential energetic compounds

Wenli Cao[1], Zimei Ding[1], Xiaojing Hang[1], Kangzhen Xu[1], Jirong Song[1, 2],
Jie Huang[1 *], Jiajia Guo[1]

[1]*School of Chemical Engineering, Shaanxi Key Laboratory of Physico-Inorganic Chemistry, Northwest University, Xi'an 710069, China*

[2]*Conservation Technology Department, the Palace Museum, Beijing 100009, China*

Contents

1. **Fig. S1.** The starting geometries extracted from the single crystal X-ray data plotted by GaussianView of **1**-**7**
2. **Fig. S2.** Optimized geometries of **1-7** at DFT-B3PW91/6-31G(d,p) level.
3. **Fig. S3.** The pictorial illustration of HOMO and LUMO at B3PW91/6-31G(d,p) level for **7**.
4. **Table S1**. The selected molecular electrostatic potential parameters of compounds **1**-**7**.
5. **Table S2**. The data of electrostatic potential parameters of ions used by densities calculation.
6. **Table S3**. The densities, detonation pressures (P) and detonation velocities (D) of **1-7** based on optimized geometries at B3PW91/6-31G(d,p) level.

*Corresponding author: E-mail: huangjie@nwu.edu.cn

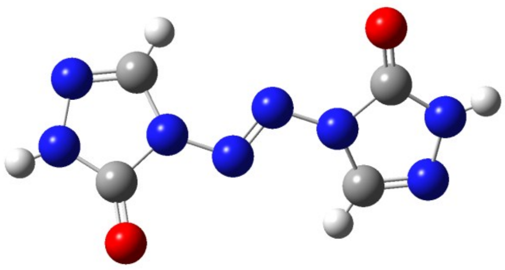

1

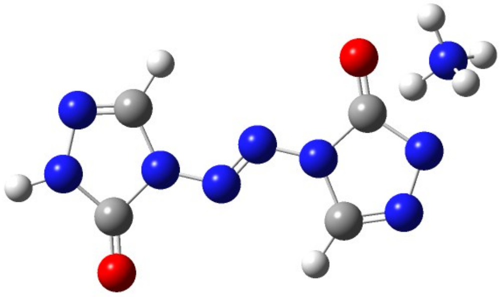

2

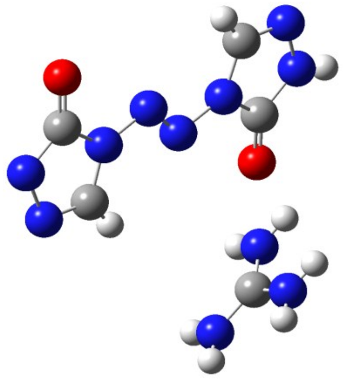

3

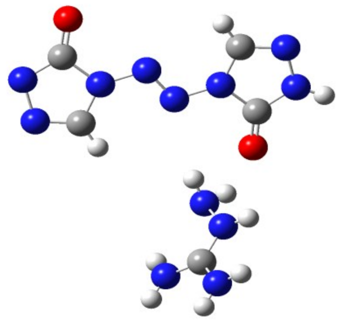

4

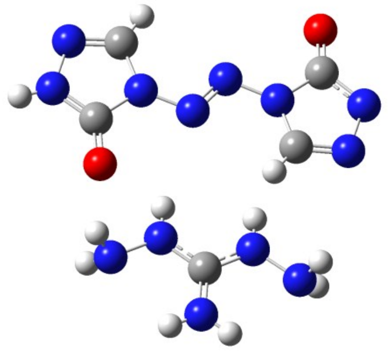

5

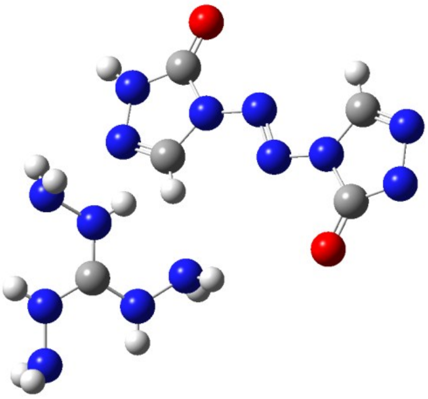

6

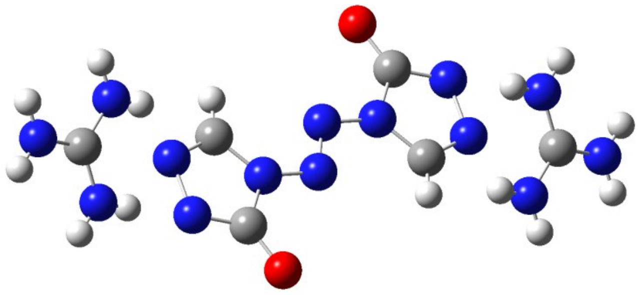

7

Fig. S1. The starting geometries extracted from the single crystal X-ray data plotted by GaussianView of **1**-**7**.

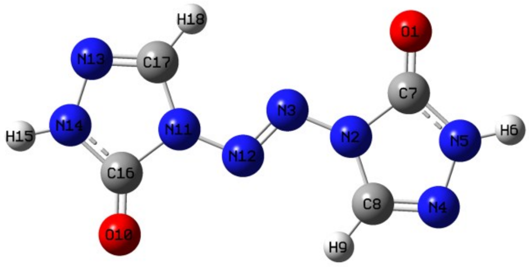

1

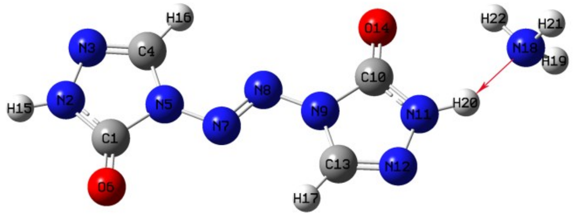

2

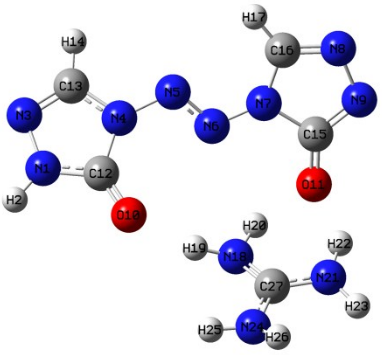

3

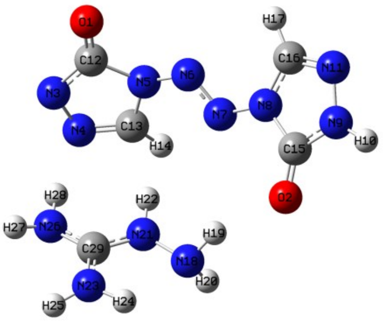

4

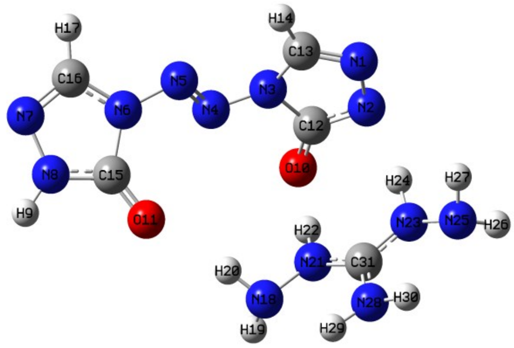

5

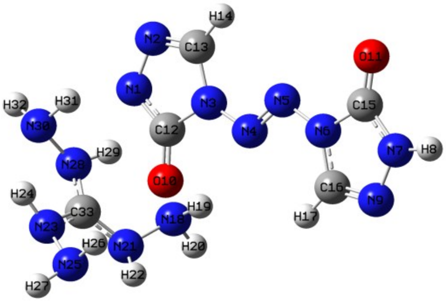

6

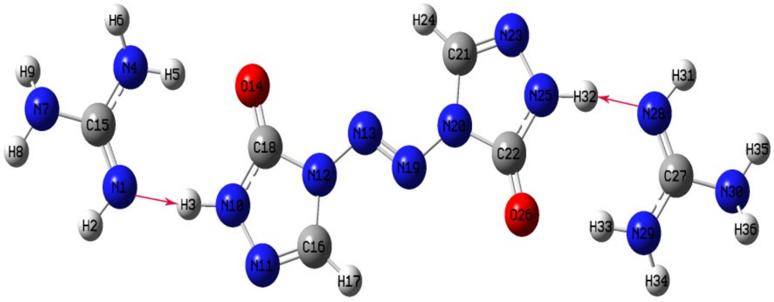

7

Fig. S2. Optimized geometries of **1-7** at DFT-B3PW91/6-31G(d,p) level.

LUMO

HOMO

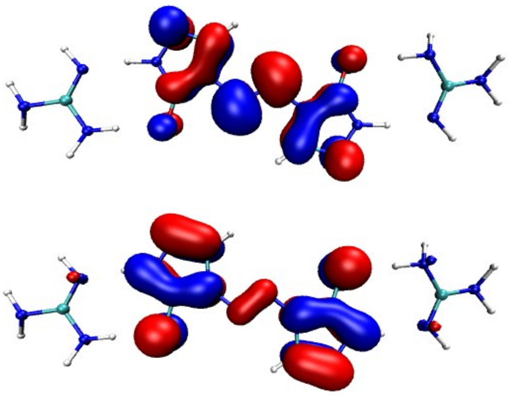

Fig. S3. The pictorial illustration of HOMO and LUMO at B3PW91/6-31G(d,p) level for **7**.

Table S1. The selected electrostatic potential parameters of compound **1-7**.

	**1**		**2**		**3**		**4**	
	B3LYP	B3PW91	B3LYP	B3PW91	B3LYP	B3PW91	B3LYP	B3PW91
A_S^+ (Å^2)	119.075	117.604	133.352	130.174	150.697	136.209	148.876	153.865
A_S^- (Å^2)	82.220	82.687	99.366	101.684	103.475	122.384	120.089	116.174

	**5**		**6**		**7**	
	B3LYP	B3PW91	B3LYP	B3PW91	B3LYP	B3PW91
A_S^+ (Å^2)	151.983	147.495	141.222	143.709	161.009[1]	130.759
A_S^- (Å^2)	134.410	138.207	155.182	155.117	158.670[1]	212.133

Table S2. The data of electrostatic potential parameters of ions used by densities calculation.

	$\overline{V}_S^+$ / kcal·mol^{-1}		$\overline{V}_S^-$ / kcal·mol^{-1}		A_S^+ / Å^2		A_S^- / Å^2	
	B3LYP	B3PW91	B3LYP	B3PW91	B3LYP	B3PW91	B3LYP	B3PW91
**2**	171.23	171.77	-81.47	-81.72	47.80	47.44	202.36	201.41
**3**	122.57	122.85	-81.47	-81.72	95.40	94.83	202.36	201.41
**4**	113.86	114.13	-81.47	-81.72	110.16	109.37	202.36	201.41
**5**	106.74	106.99	-81.47	-81.72	125.16	124.18	202.36	201.41
**6**	101.02	101.23	-81.47	-81.72	139.87	138.71	202.36	201.41

Table S3. The densities, detonation pressures (P) and detonation velocities (D) of 1-7 based on optimized geometries at B3PW91/6-31G(d,p) level.

	**1**	**2**	**3**	**4**	**5**	**6**	**7**
$\rho_{cal}{}^{a}$/ g·cm^{-3}	1.775	1.816	1.680	1.668	1.659	1.653	1.640
P^{b}/ GPa	22.37	27.07	20.94	21.85	22.80	23.71	18.06
D^{c}/ ms^{-1}	7131	7792	7107	7185	7352	7505	6568

^{a} Calculated density. ^{b} Detonation pressure. ^{c} Detonation velocity.

1. W. L. Cao, J. J. Guo, X. Chen, Z. M. Ding, K. Z. Xu, J. R. Song, A. Fan and J. Huang, *J. Mol. Struct.*, 2017, 1147, 754-762.
